# Infrared-Assisted Temperature-Aware Backscatter Access for UAV-Enabled Geothermal Hotspot Sensing

**DOI:** 10.3390/s26051686

**Published:** 2026-03-06

**Authors:** Chong Li, Yuxiang Cheng, Siqing He, Zhenxing Li

**Affiliations:** 1 Hebei Key Laboratory of Precision Exploration Technology for Deep Geothermal Resources, Xiongan New Area 070001, China; 2 Hydrogeology Bureau of China National Administration of Coal Geology, Xiongan New Area 070001, China; 3 College of New Energy and Environment, Jilin University, Changchun 130015, China

**Keywords:** geothermal hotspot sensing, UAV-enabled IoT, wireless-powered backscatter, thermal infrared imaging, temperature-aware medium access

## Abstract

Geothermal exploration and monitoring often require dense temperature observations in terrains where wired networks are impractical and battery replacement for in situ sensors is costly. This paper proposes an infrared-assisted, temperature-aware access scheme for a UAV-enabled backscatter IoT network tailored to geothermal hotspot sensing. A rotary-wing UAV equipped with a thermal infrared camera and an RF transceiver first surveys the area to construct a surface temperature map and identify candidate hotspots, and then hovers above a selected hotspot to perform periodic frames consisting of wireless energy transfer followed by backscatter uplink collection. Ground sensors harvest RF energy, measure their local temperature, and autonomously activate only when both the harvested energy exceeds a threshold and the measured temperature falls within a target interval broadcast by the UAV, thereby concentrating channel access on thermally relevant nodes. We develop a system model that couples a geothermal-like thermal field, RF energy harvesting, and framed slotted backscatter access, and introduce hotspot-oriented performance metrics including effective hotspot throughput, task completion time, and energy per hotspot report. The simulation results show that the proposed temperature–energy-gated access significantly increases the fraction of successfully decoded packets originating from hotspot regions and improves the energy efficiency of geothermal monitoring compared with full activation and purely energy-based activation.

## 1. Introduction

Geothermal resources such as hot springs, fumaroles, and shallow hydrothermal systems play an increasingly important role in regional clean-energy portfolios and hazard monitoring. In anomalously high-heat-flow regions, geothermal systems transfer heat from depth to the Earth’s surface, often forming localized thermal anomalies that can be detected from surface temperature measurements and thermal infrared (TIR) remote sensing. Compared with traditional geophysical prospecting methods based on dense field surveys and well logging, TIR remote sensing provides a convenient and cost-effective way to delineate geothermal prospective areas and map land surface temperature (LST) anomalies over large regions. Recent advances in satellite- and UAV-borne TIR sensors have enabled fine-scale mapping of geothermal manifestations and surface heat flux, including diffuse thermal water discharge and cryptic fumarolic activity, which are difficult to detect from visible imagery alone.

While TIR remote sensing is powerful for locating candidate geothermal anomalies at the surface, it is less suited for continuous, in situ monitoring of temperature evolution and for capturing small-scale spatial variability near faults, fractures, and engineered structures. To complement remote sensing, many geothermal studies deploy distributed temperature sensing networks or arrays of point sensors to record subsurface or near-surface temperature fields at high temporal resolution. However, installing and maintaining wired networks in rugged geothermal terrains is costly, and battery replacement for dense wireless sensor deployments is often impractical. These constraints make ultra-low-power, wirelessly powered Internet-of-Things (IoT) architectures highly attractive for geothermal monitoring, especially in early-stage exploration or long-term surveillance of remote fields.

Ambient and wireless-powered backscatter communication networks have emerged as promising techniques to support such battery-less or battery-constrained sensing nodes. In backscatter systems, devices transmit data by modulating the reflection of incident radio-frequency (RF) signals using a simple impedance-switching circuit, avoiding active RF chains and enabling operation with only a few microwatts of power harvested from the environment. Recent surveys on ambient backscatter and hybrid wireless-powered backscatter networks highlight their potential for large-scale IoT deployments where energy efficiency and hardware simplicity are paramount. Parallel research on UAV-enabled wireless-powered communication networks (WPCNs) and backscatter systems shows that UAVs can serve as mobile RF energy sources and data collectors, with optimized trajectories and resource allocation significantly improving throughput and energy efficiency for ground sensor nodes.

Despite these advances, most UAV-assisted WPCN and backscatter designs remain application-agnostic. They typically treat all energized sensors as homogeneous contenders and optimize global metrics such as total throughput, coverage probability, or network-wide energy efficiency. In geothermal monitoring, this assumption is often sub-optimal because only sensors located within or near thermal anomalies (e.g., hot faults, steaming grounds, or elevated-LST patches) provide mission-critical information. By contrast, nodes in thermally normal regions contribute little to anomaly characterization while still consuming channel opportunities and harvested energy. When all energized nodes contend, collisions increase and RF energy is wasted, and the fraction of successfully received packets that originate from hot zones can remain small. Existing temperature-aware protocols in sensor networks mainly use temperature for routing or load balancing in battery-powered systems, and they do not address UAV-borne RF powering, passive backscatter access, or infrared-guided hotspot selection.

This research gap motivates an application-oriented access design that closes the loop between thermal reconnaissance and in situ data acquisition. The UAV first performs a coarse TIR scan to delineate thermally anomalous regions and selects a target hotspot. It then hovers above the hotspot center and executes periodic frames comprising an RF energy-transfer phase followed by a slotted backscatter uplink phase. At the beginning of each frame, the UAV transmits RF energy and broadcasts lightweight control information, including a target temperature window for the selected hotspot. Each sensor locally evaluates a joint condition on harvested energy and measured temperature, and it contends for channel access only when both conditions are satisfied. This TIR-derived temperature window, coupled with energy gating, reduces the contention set and prioritizes thermally relevant nodes, thereby improving hotspot packet yield and energy efficiency.

In this work, we develop an infrared-assisted, temperature-aware UAV-based sensing framework for geothermal hotspot monitoring using low-power backscatter sensor nodes. The UAV employs onboard thermal infrared (TIR) imaging to obtain a coarse thermal map of the monitored area and to identify regions exhibiting elevated land surface temperature (LST). It then hovers over a selected hotspot and periodically broadcasts radio-frequency (RF) energy together with lightweight control packets specifying the frame configuration and a target temperature window. Ground sensors harvest energy from the RF signal, measure their local temperature, and participate in framed slotted backscatter transmission only when both their harvested energy and temperature satisfy the broadcast criteria; otherwise, they remain in a low-power sleep state. To characterize sensing performance on geothermal anomalies, we define the effective hotspot throughput as the average number of successfully decoded packets per frame originating from sensors whose ground-truth temperatures fall within the target window. Based on this metric, we further evaluate the hotspot task completion time and the energy cost per effective hotspot packet, which jointly reflect the efficiency and selectivity of UAV-assisted geothermal monitoring.

The main contributions of this paper are summarized as follows:

(1) We formulate a hotspot-oriented UAV-assisted sensing problem that couples a geothermal-like temperature field, UAV-borne TIR reconnaissance, RF energy transfer, and slotted backscatter access, enabling task-driven performance evaluation beyond conventional throughput-centric metrics.

(2) We propose a lightweight external-prior-driven activation mechanism, where a UAV-broadcast temperature window derived from TIR mapping is jointly applied with an energy threshold to gate node participation. This shrinks the contention set toward thermally relevant sensors without per-node scheduling or heavy signaling.

(3) We introduce hotspot-oriented metrics, including effective hotspot throughput, hotspot task completion time, and energy cost per effective hotspot packet, and develop a Monte Carlo evaluation framework to quantify the selectivity–efficiency trade-off compared with full activation and energy-only activation baselines.

The proposed temperature–energy gating differs from prior temperature-aware designs that use temperature mainly for routing or load balancing, and from generic wireless-powered backscatter access that targets overall throughput. In our setting, TIR mapping is explicitly translated into a broadcast temperature window that gates MAC-layer participation and prioritizes hotspot-relevant sensing.

The remainder of this paper is organized as follows. Section 2 introduces the related works. Section 3 describes the proposed mechanism. Section 4 presents the numerical results and discusses design implications for geothermal exploration and monitoring, and Section 5 concludes the paper and outlines directions for extending the framework with optimization-based configuration and joint UAV trajectory and access control.

## 2. Related Works

This section briefly reviews three lines of research related to our work: (i) thermal infrared (TIR) remote sensing and geothermal hotspot mapping, (ii) battery-less IoT and UAV-enabled backscatter networks, and (iii) temperature-aware protocols in low-power sensor networks. We then highlight the research gap that motivates the proposed temperature-aware, energy-gated backscatter access scheme for geothermal sensing.

Geothermal hotspot detection and TIR remote sensing: Thermal infrared (TIR) remote sensing has become a core technique for identifying geothermal prospective areas and characterizing surface heat-flow anomalies over large spatial scales. Early and recent studies have demonstrated that land surface temperature (LST) anomalies derived from satellite TIR sensors, such as Landsat, MODIS, ASTER, and ECOSTRESS, provide an effective proxy for shallow geothermal activity when combined with appropriate inversion and correction strategies. Comprehensive reviews have systematically summarized the progress of TIR-based geothermal exploration, highlighting advances in surface temperature inversion algorithms, anomaly extraction, and the integration of geological, geophysical, and geochemical constraints to improve interpretability and reliability [1].

A large body of recent work has focused on multi-temporal and multi-source TIR analysis to suppress environmental disturbances and enhance geothermal signals. For example, Li et al. employed long-term winter LST time series with terrain and altitude corrections to extract stable geothermal anomalies in the Qinghai–Tibet Plateau, demonstrating that winter mean LST is particularly sensitive to subsurface heat sources [2]. Similar multi-temporal strategies using Landsat 8 have been adopted in tectonically controlled basins, where persistent high-LST regions were shown to align with fault systems acting as thermal conduits [3]. Multi-view day–night fusion approaches further improved anomaly discrimination by reducing non-geothermal thermal effects, as demonstrated through Dempster–Shafer evidence theory and fault-buffer analysis [4,5].

Beyond purely thermal indicators, several studies integrated TIR data with complementary proximal or geochemical measurements. Sun et al. combined Landsat-derived LST anomalies with soil gas radon surveys to delineate blind geothermal targets beneath dense vegetation, successfully guiding geothermal drilling [6]. Rabuffi et al. adopted a multiscale framework that jointly analyzed satellite LST, DEM-derived structural lineaments, and UAV-based high-resolution thermal imagery, revealing how brittle tectonics governs the spatial distribution of geothermal surface manifestations [7]. These works underscore the importance of coupling TIR observations with structural interpretation to infer geothermal fluid pathways.

At finer spatial scales, UAV-borne TIR sensors have increasingly been used to capture localized thermal anomalies that are difficult to resolve using satellite data alone. High-resolution thermal maps obtained from drone-mounted FLIR cameras have been applied to fumarolic fields, volcanic slopes, and diffuse hot-ground zones, enabling the detection of meter-scale temperature variations and short-term thermal dynamics [8]. Related studies also explored thermal downscaling techniques and spatiotemporal fusion methods to bridge the resolution gap between satellite and airborne observations, further enhancing geothermal anomaly mapping capabilities [9].

Despite these advances, existing TIR-based geothermal studies primarily treat thermal imagery as a passive observation tool. Thermal maps are typically used for anomaly delineation, heat-flow estimation, or tectonic interpretation, but they rarely influence the behavior of in situ sensor networks or subsequent data acquisition processes. In particular, the potential of TIR-detected hotspots to actively guide sensor activation, communication scheduling, or UAV-assisted data collection has not been sufficiently explored. This research gap motivates the present work, which integrates TIR-driven hotspot detection with UAV-enabled, temperature-aware interaction and backscatter-based data acquisition, bridging thermal remote sensing and low-power IoT system design.

Battery-less IoT and ambient backscatter communication: Battery-less Internet of Things (IoT) enabled by ambient and wireless-powered backscatter communication has emerged as a promising paradigm for large-scale, maintenance-free sensing deployments. Comprehensive surveys have summarized the fundamental principles of backscatter modulation, system architectures, and key challenges, including extremely weak reflected signals, a short communication range, and severe interference [10]. By leveraging existing ambient radio-frequency (RF) sources such as television, cellular, and Wi-Fi signals, backscatter devices can operate with only microwatts of harvested power, making them attractive for dense and long-term monitoring scenarios where battery replacement is impractical.

To overcome the limited coverage and reliability of ground-based backscatter systems, the integration of unmanned aerial vehicles (UAVs) has received increasing attention in recent years. UAVs can serve as mobile RF power beacons, data collectors, or hybrid access points, significantly enhancing communication range and energy availability. A series of analytical and optimization-based studies have investigated UAV-enabled backscatter communication systems by jointly designing UAV trajectories, hovering positions, time allocation, and reflection coefficients. For instance, Wu et al. optimized user scheduling, transmit power, and UAV trajectories to maximize the achievable rate of ambient backscatter users [11]. Du et al. further incorporated non-orthogonal multiple access (NOMA) into UAV-aided backscatter networks, enabling simultaneous data collection from multiple devices and achieving notable throughput gains through joint trajectory and reflection coefficient optimization [12].

Beyond throughput maximization, several works have focused on energy efficiency, timeliness, and system-level trade-offs. Long et al. studied long-term average AoI minimization in a multi-UAV-assisted backscatter sensing network with NOMA forwarding, by jointly optimizing the GUs’ access control, the UAVs’ trajectories, and beamforming via a Lyapunov optimization framework. [13]. investigated joint laser-assisted wireless powering and data collection in UAV-aided backscatter sensor networks, addressing the laser energy consumption–battery retention trade-off through an alternating optimization framework [14]. These studies demonstrate that UAV mobility can substantially improve both communication performance and energy sustainability.

Related research has also explored UAV-enabled data collection and wireless power transfer in broader IoT contexts. Liu et al. optimized three-dimensional UAV placement and resource allocation to minimize the total transmission power of IoT devices under quality-of-service constraints [15]. Fu et al. and Dandapat et al. considered joint UAV trajectory/route design and energy–time trade-offs in UAV-assisted IoT networks, aiming to balance task completion/service time and UAV energy consumption under practical energy constraints [16,17]. In addition, Yoon proposed adaptive data acquisition control schemes to mitigate energy imbalance in wireless rechargeable sensor networks using UAVs as mobile sinks [18].

Temperature-aware protocols in low-power sensor networks: Temperature has been investigated as a protocol-level metric in WBANs and other low-power sensor networks. A line of works incorporates temperature into routing decisions (i.e., temperature-aware routing) together with energy and QoS considerations to improve network operation under the constraints of body sensor nodes [19].

Subsequent studies further integrate temperature awareness with energy-efficient route selection, congestion control, trust evaluation, and link-quality optimization to jointly improve thermal distribution and communication reliability [20,21,22,23]. In these protocols, temperature is inherently tied to the operation of actively transmitting nodes and is used to regulate intra- or inter-body routing behavior under strict power and safety constraints.

Limited work has jointly integrated (i) UAV-borne TIR imaging for geothermal hotspot detection, (ii) RF-powered backscatter communications for battery-less ground sensors, and (iii) temperature-aware medium-access activation driven by an external environmental field. Existing geothermal TIR studies mainly focus on hotspot delineation and interpretation, but they rarely close the loop to regulate in situ sensing or communication behaviors. Existing UAV-enabled backscatter/WPCN frameworks optimize trajectory and resource allocation, yet they typically assume application-agnostic access where all energized nodes act as homogeneous contenders and performance is evaluated by global throughput/outage rather than hotspot information yield. Temperature-aware protocols in WBSNs/WSNs primarily regulate routing or self-heating safety for battery-powered active radios, instead of gating uplink access in a wireless-powered backscatter MAC.

In contrast, our work introduces an external-prior-driven access control principle: TIR-derived hotspot signatures are summarized into a broadcast temperature window and coupled with energy gating to shrink the contention set in the temperature domain. We further evaluate the system using hotspot-oriented metrics (effective hotspot throughput, hotspot task completion time, and energy per hotspot report), which directly reflect geothermal monitoring objectives rather than generic network throughput.

## 3. System Model

**Figure 1:**
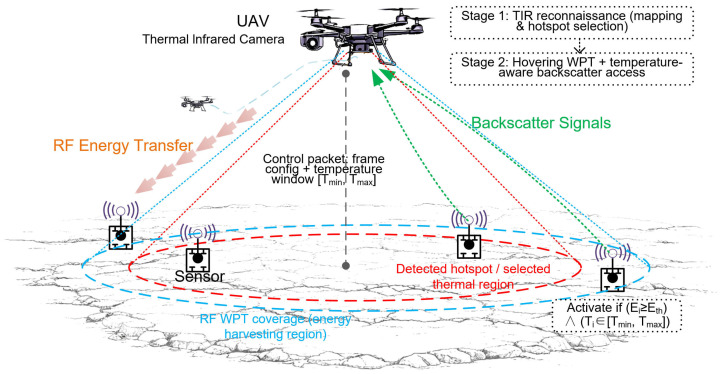
System framework.

In this section, we present the system model of the proposed infrared-assisted, temperature-aware backscatter access scheme for geothermal hotspot sensing. We first describe the network geometry and geothermal thermal field, then introduce the infrared-based hotspot selection stage. Based on this, we model the RF energy-harvesting process, the framed backscatter uplink, and the joint temperature–energy activation rule that shrinks the contention set around the hotspot of interest (see Figure 1).

### 3.1. Network Geometry and Geothermal Thermal Field

We consider a geothermal field represented by a two-dimensional region $A\subset R^{2}$ with area $\left|A\right|=L^{2}$. A set of (N) battery-less sensing nodes are deployed over A. Unless otherwise stated, we assume that node locations $u_{i}=\left(x_{i},y_{i}\right),i=1,\ldots ,N$ are independent and identically distributed (i.i.d.) and follow a uniform distribution over A. The nodes are installed close to the ground surface and are equipped with a temperature sensor that measures the local surface (or near-surface) temperature; a simple RF energy-harvesting circuit; and a backscatter modulator controlled by a low-power microcontroller. To capture geothermal heterogeneity, we model the surface temperature as a superposition of a background temperature field and (K) geothermal hotspots. Let $c_{k}=\left(x_{k}^{\left(h\right)},y_{k}^{\left(h\right)}\right)$ denote the location of hotspot *k*, and $\Delta T_{k}>0$ its peak temperature elevation above the background. A commonly used phenomenological model is the sum of Gaussian-shaped anomalies:

(1) $$T\left(x,y\right)=T_{\mathrm{bg}}\left(x,y\right)+\sum_{k=1}^{K}\Delta T_{k}\exp\;\left(-\frac{{∥\left(x,y\right)-c_{k}∥}^{2}}{2\sigma_{k}^{2}}\right),$$

where $T_{\mathrm{bg}}\left(x,y\right)$ is the slowly varying background temperature and $\sigma_{k}$ characterizes the spatial extent of hotspot (k). In many practical geothermal applications, the background variation over a local area of interest is modest, and we can approximate $T_{\mathrm{bg}}\left(x,y\right)\approx T_{0}$ as a constant reference temperature. Then the local temperature at node (i) is

(2) $$T_{i}≜T\left(x_{i},y_{i}\right),\;i=1,\ldots ,N.$$

A node is considered geothermally relevant to a specific hotspot sensing task if its temperature lies within a target interval $\left[T_{\min},T_{\max}\right]$ associated with the anomaly of interest. This set will later be used to define “hotspot-effective” performance metrics.

A rotary-wing unmanned aerial vehicle (UAV) operates above the geothermal field. The UAV is equipped with (i) a downward-looking thermal infrared (TIR) camera and (ii) an RF transceiver that acts as both a continuous-wave (CW) energy source and a backscatter reader. During data collection, the UAV hovers at a fixed altitude (H) above the ground at position $q=(x_{q},y_{q},H)$. For simplicity and without loss of generality, we assume that, after hotspot selection (Section 3.2), the UAV hovers above the horizontal center of the target hotspot region.

### 3.2. Infrared-Assisted Hotspot Selection

The sensing task is organized into two conceptual stages: an infrared reconnaissance stage and a backscatter access stage. In the reconnaissance stage, the UAV flies over A at an altitude of $H_{\mathrm{IR}}$ and collects a sequence of TIR images. From these images, it constructs an estimated surface temperature map $\hat{T}\left(x,y\right)$ over the region. Standard TIR processing techniques (radiometric calibration, atmospheric and emissivity correction, terrain normalization) are assumed to have been applied by the onboard or ground-based processing system.

Using $\hat{T}\left(x,y\right)$, the UAV identifies candidate hotspot regions as connected components of the suprathreshold set

(3) $$\hat{H}\left(\theta_{\mathrm{IR}}\right)=\left\{\left(x,y\right)\in A|\hat{T}\left(x,y\right)-{\hat{T}}_{\mathrm{bg}}\left(x,y\right)\geq \theta_{\mathrm{IR}}\right\},$$

where $\theta_{\mathrm{IR}}$ is a user-selected anomaly threshold in Kelvin or degrees Celsius, and ${\hat{T}}_{\mathrm{bg}}\left(x,y\right)$ is an estimated background temperature. For a given sensing mission, the control center selects one hotspot region $H_{0}\subset \hat{H}\left(\theta_{\mathrm{IR}}\right)$ as the primary target, for example based on its area, peak intensity or proximity to critical infrastructure.

In the second stage, which is the focus of this paper, the UAV moves to the geometric center of $H_{0}$, denoted by $c_{0}$, and hovers at altitude *H*. While hovering, it repeatedly executes energy-transfer and backscatter-collection frames over a mission horizon $T_{\mathrm{mission}}$. The hotspot information obtained from TIR reconnaissance is summarized as a target temperature window $\left[T_{\min},T_{\max}\right]$ that characterizes the thermal signature of $H_{0}$, and this window is broadcast to ground nodes as part of a lightweight control packet at the beginning of each frame.

In the following analysis, we abstract the TIR processing stage and assume that the hotspot center $c_{0}$ and the target temperature window $\left[T_{\min},T_{\max}\right]$ are obtained from standard TIR mapping. These parameters are treated as given and remain fixed during the backscatter access stage.

### 3.3. RF Energy-Harvesting Model

We adopt a standard wireless power transfer model to describe the RF energy-harvesting process. During the wake-up phase of each frame, the UAV transmits a continuous-wave (CW) RF signal with power $P_{t}$ at carrier frequency $f_{c}$. The complex baseband channel between the UAV and node *i* is denoted by $h_{i}$, and the corresponding large-scale path loss is modeled as

(4) $$L\left(d_{i}\right)=\beta_{0}\;d_{i}^{-\alpha },$$

where $d_{i}=\sqrt{{∥u_{i}-c_{0}∥}^{2}+H^{2}}$ is the 3D distance between the UAV and node *i*, α≥2 is the path-loss exponent, and $\beta_{0}$ is the reference gain at 1 m. The average received RF power at node *i* is then

(5) $$P_{i}^{\left(\mathrm{rx}\right)}=P_{t}L\left(d_{i}\right).$$

To explicitly capture the benefit of hovering above the hotspot, we distinguish between the horizontal distance of node *i* to the hotspot center,

$$\rho_{i}=∥\left(x_{i},y_{i}\right)-c_{0}∥,$$

and the 3D distance

$$d_{i}=\sqrt{\rho_{i}^{2}+H^{2}}.$$

Assuming that geothermal hotspot nodes lie within a disk of radius $r_{h}$ around $c_{0}$, i.e., $\rho_{i}\leq r_{h}$, whenever $T_{i}\in \left[T_{\min},T_{\max}\right]$, their distances satisfy

(6) $$H\leq d_{i}^{\left(\mathrm{hot}\right)}\leq \sqrt{r_{h}^{2}+H^{2}}.$$

By contrast, under a baseline design where the UAV hovers above a generic point in the field, hotspot nodes are spread over a larger disk of radius $R>r_{h}$, yielding

(7) $$H\leq d_{i}^{\left(\mathrm{base}\right)}\leq \sqrt{R^{2}+H^{2}}.$$

Since the harvested energy in (9) scales as $E_{i}\propto d_{i}^{-\alpha }$, hovering above the hotspot yields

(8) $$E\;\left[E_{i}^{\left(\mathrm{hot}\right)}\right]>E\;\left[E_{i}^{\left(\mathrm{base}\right)}\right]$$

for hotspot nodes, which directly increases their energy activation probability and facilitates subsequent backscatter transmission.

Each node is equipped with a rectifying antenna (rectenna) whose RF-to-DC conversion efficiency is denoted by $\eta_{\mathrm{EH}}\in \left(0,1\right]$. Let $\tau_{w}$ denote the duration of the wake-up phase in each frame. The harvested energy at node *i* during one wake-up phase is approximated as

(9) $$E_{i}=\eta_{\mathrm{EH}}P_{t}L\left(d_{i}\right)\tau_{w}.$$

A minimum amount of energy $E_{\mathrm{th}}$ is required to (i) power the sensing and baseband circuitry, (ii) perform one temperature measurement, and (iii) drive the backscatter switch and control logic during the subsequent backscatter slot. We model this using an energy activation indicator:

(10) $$I_{i}^{\left(E\right)}=\left\{\begin{array}{cc} 1, & E_{i}\geq E_{\mathrm{th}},\\ 0, & E_{i}<E_{\mathrm{th}}. \end{array}\right.$$

Hence, only nodes with $I_{i}^{\left(E\right)}=1$ are eligible to participate in uplink backscatter within that frame.

It is sometimes convenient to work with the energy activation probability $p_{E}$ rather than the deterministic indicator. Assuming that node positions are i.i.d. uniformly distributed over a disk of radius *R* around $c_{0}$, the horizontal distance $\rho_{i}=∥\left(x_{i},y_{i}\right)-c_{0}∥$ admits a closed-form density $f_{\rho }\left(\rho \right)$.

(11) $$p_{E}=\mathrm{Pr}\left\{E_{i}\geq E_{\mathrm{th}}\right\}=\mathrm{Pr}\left\{\rho_{i}\leq \rho_{\max}\right\}=\int_{0}^{\rho_{\max}}f_{\rho }\left(\rho \right)\;d\rho ,$$

where $\rho_{\max}=\sqrt{d_{\max}^{2}-H^{2}}$ is the corresponding maximum horizontal distance, and $d_{\max}$ is the maximum 3D distance at which the harvested energy meets the threshold, obtained by inverting (9):

(12) $$d_{\max}={\left(\frac{\eta_{\mathrm{EH}}P_{t}\tau_{w}\beta_{0}}{E_{\mathrm{th}}}\right)}^{1/\alpha }.$$

This probabilistic view is useful when deriving the expected size of the active set.

### 3.4. Backscatter Uplink and Frame Structure

Each frame is divided into two phases: an RF energy-transfer (wake-up) phase of length $\tau_{w}$ and a backscatter access phase of length $\tau_{b}$. The latter is further partitioned into *M* equal-duration slots of length $\delta =\tau_{b}/M$. At the beginning of the frame, the UAV broadcasts a short control packet that includes the frame configuration $\left(M,\delta ,\tau_{w}\right)$ and the target temperature window $\left[T_{\min},T_{\max}\right]$.

In the backscatter phase, each *eligible* node *i* that passed the energy and temperature checks (specified in Section 3.5) contends for the channel using a framed slotted ALOHA mechanism. Specifically, the node independently and uniformly chooses one slot index $s_{i}\in \left\{1,\ldots ,M\right\}$ and backscatters its data only in that slot. Let U⊆{1,…,N} denote the set of active nodes in the current frame, and let

(13) $$U_{m}=\left\{i\in U|s_{i}=m\right\}$$

be the subset of nodes transmitting in slot *m*. The UAV receives the aggregate backscattered signal in each slot. Assuming a single-tone CW excitation with power $P_{t}$, the received baseband signal in slot *m* can be written as

(14) $$y_{m}\left(t\right)=\sum_{i\in U_{m}}\sqrt{P_{t}L\left(d_{i}\right)}\;g_{i}\;x_{i}\left(t\right)+n_{m}\left(t\right),\;0\leq t\leq \delta ,$$

where $g_{i}$ is the effective backscatter channel coefficient from node *i* (including forward and reverse paths and the reflection coefficient), $x_{i}\left(t\right)$ is the unit-power baseband modulation waveform of node *i*, and $n_{m}\left(t\right)$ is additive noise.

For a monostatic backscatter link, the effective large-scale attenuation involves a double path-loss term. A simple model for the average received signal-to-noise ratio (SNR) of node *i* in a singleton slot is

(15) $$\gamma_{i}=\frac{P_{t}\;\beta_{\mathrm{bs}}^{2}\;d_{i}^{-2\alpha }}{N_{0}B},$$

where $\beta_{\mathrm{bs}}$ lumps together antenna gains and reflection coefficients, $N_{0}$ is the noise spectral density, and *B* is the receiver bandwidth. The decoding success probability in a singleton slot can then be expressed as

(16) $$p_{\mathrm{dec}}\left(d_{i}\right)=\mathrm{Pr}\left\{\gamma_{i}\geq \gamma_{\mathrm{th}}\right\},$$

with $\gamma_{\mathrm{th}}$ denoting the SNR threshold of the backscatter demodulator. Because $\gamma_{i}\propto d_{i}^{-2\alpha }$ in (15), reducing the UAV–sensor distance for hotspot nodes by hovering above $c_{0}$ yields

(17) $$E\;\left[\gamma_{i}^{\left(\mathrm{hot}\right)}\right]>E\;\left[\gamma_{i}^{\left(\mathrm{base}\right)}\right],$$

and thus a higher average decoding probability for packets originating from hotspot nodes.

In this work we focus on the MAC-layer contention behavior rather than detailed physical-layer detection algorithms. We adopt a standard collision model: (i) if $|A_{m}|\;=0$, slot *m* is idle; (ii) if $|A_{m}|\;=1$, the UAV can decode the packet from the unique node with probability $p_{\mathrm{dec}}$; (iii) if $|A_{m}|\;\geq 2$, a collision occurs and none of the packets in slot *m* are successfully decoded. Under this model, the number of successfully decoded packets in a frame is

(18) $$S=\sum_{m=1}^{M}I\left(|A_{m}|\;=1\right)\cdot Z_{m},$$

where I(·) is the indicator function and $Z_{m}$ is a Bernoulli random variable capturing the decoding success in a singleton slot (equal to 1 with probability $p_{\mathrm{dec}}$).

### 3.5. Temperature-Aware Energy-Gated Activation

The key mechanism that differentiates the proposed scheme from conventional energy-harvesting backscatter MACs is the combination of *energy gating* and *temperature gating* driven by the TIR-derived hotspot information. At the beginning of each frame, after the wake-up phase and local temperature measurement, node *i* evaluates two conditions:

1. Energy condition:
  (19) $$E_{i}\geq E_{\mathrm{th}}\;\Leftrightarrow \;I_{i}^{\left(E\right)}=1.$$
2. Temperature condition:
  (20) $$T_{\min}\leq T_{i}\leq T_{\max}\;\Leftrightarrow \;I_{i}^{\left(T\right)}=1,$$
  where $I_{i}^{\left(T\right)}$ is a temperature activation indicator.

Only nodes that satisfy both conditions are allowed to contend for channel access in that frame. Formally, the activation indicator for node *i* is

(21) $$I_{i}^{\left(\mathrm{act}\right)}=I_{i}^{\left(E\right)}\cdot I_{i}^{\left(T\right)}=\left\{\begin{array}{cc} 1, & E_{i}\geq E_{\mathrm{th}}\;\mathrm{and}\;T_{\min}\leq T_{i}\leq T_{\max},\\ 0, & \mathrm{otherwise}. \end{array}\right.$$

The active node set is

(22) $$A=\{i\in \left\{1,\ldots ,N\right\}|I_{i}^{\left(\mathrm{act}\right)}=1\},$$

with cardinality K=|A|. Intuitively, the energy condition restricts activations to nodes lying within a WPT-effective region around the UAV, while the temperature condition further shrinks the contention set to nodes located within the geothermal hotspot (or its thermal signature) indicated by TIR.

For analytical insight, it is useful to approximate the average size of A. Let $p_{E}$ denote the energy activation probability in (11), and let

(23) $$p_{T}=\mathrm{Pr}\left\{T_{\min}\leq T_{i}\leq T_{\max}\right\}={\int \int }_{(x,y)\in A}I\;\left(T\left(x,y\right)\in \left[T_{\min},T_{\max}\right]\right)\frac{1}{\left|A\right|}\;dxdy$$

be the probability that a randomly placed node lies within the target temperature window. If we assume that the energy and temperature conditions are approximately independent (which is reasonable when the WPT-effective region covers the hotspot), the expected number of active nodes can be approximated as

(24) $$E\left[K\right]\approx N\;p_{E}\;p_{T}.$$

This expression highlights the *set-shrinking* effect of the proposed mechanism: compared with a full activation scheme ($p_{E}=p_{T}=1$) or a purely energy-based scheme ($p_{T}=1$), the joint temperature–energy gating reduces the contention set size by a factor of $p_{T}$, concentrating channel access on nodes whose temperature is consistent with the geothermally anomalous region.

To explicitly link the activation rule to geothermal sensing performance, we define a hotspot indicator for node *i*,

(25) $$I_{i}^{\left(H\right)}=\left\{\begin{array}{cc} 1, & T_{i}\in \left[T_{\min},T_{\max}\right],\\ 0, & \mathrm{otherwise}, \end{array}\right.$$

and decompose the total number of successful packets *S* in (18) into

(26) $$S=S_{H}+S_{N},$$

where $S_{H}$ and $S_{N}$ denote the numbers of successful packets originating from hotspot nodes ($I_{i}^{\left(H\right)}=1$) and non-hotspot nodes ($I_{i}^{\left(H\right)}=0$), respectively. The *effective hotspot throughput* is then defined as

(27) $$\Theta_{H}≜\frac{E[S_{H}]}{\tau_{w}+\tau_{b}},$$

which will be used in the performance evaluation to quantify how efficiently the system collects geothermal anomaly information.

Combining (8) and (17), the infrared-guided hovering strategy simultaneously increases the harvested energy and backscatter SNR of hotspot nodes compared with a baseline hovering point, which boosts both their activation probability and their decoding probability. As a result, the proposed temperature-aware, energy-gated activation not only shrinks the contention set around the hotspot via $p_{T}$, but also operates under more favorable channel conditions for the targeted sensors.

## 4. Simulation Results and Discussion

We evaluate the proposed infrared-assisted, temperature-aware backscatter access scheme through Monte Carlo simulations. All results are averaged over $N_{\mathrm{run}}=200$ independent realizations of the node deployment and temperature field. A square geothermal field of size L×L is considered, where L=200 m. A single geothermal hotspot is located at the center of the area and modeled by the Gaussian temperature field in Section 3.1 with standard deviation $\sigma_{h}$ and peak elevation $\Delta T_{h}$. Ground sensor nodes are deployed uniformly at random with density $\lambda =N/L^{2}$, where the total number of nodes *N* varies from 100 to 400. Each node measures its local surface temperature and is classified as a *hotspot node* if its temperature lies within the target interval $\left[T_{\min},T_{\max}\right]$ derived from the infrared map.

The UAV hovers at altitude *H* directly above the hotspot center. During each frame, it first transmits a continuous-wave signal for a duration of $\tau_{w}$ to charge the nodes, and then runs a framed backscatter uplink with *M* time slots of length δ. The RF channel follows the large-scale path-loss model $L\left(d\right)=\beta_{0}d^{-\alpha }$ with path-loss exponent α=2.2 and reference gain $\beta_{0}$, and the harvested energy at node *i* is given by (9). The RF-to-DC conversion efficiency is set to $\eta_{\mathrm{EH}}=0.5$, and the energy threshold required for activation $E_{\mathrm{th}}$ is chosen such that nodes within a radius of about 60–80 m around the UAV can be activated with high probability.

In the backscatter phase, each active node independently chooses one of the M=16 slots and backscatters a fixed-length packet. We adopt the collision model in Section 3.4 with decoding success probability $p_{\mathrm{dec}}=0.95$ in singleton slots. Three benchmark activation schemes are considered:

- Full activation: All *N* nodes contend in every frame, i.e., no energy or temperature gating is applied.
- Energy-only activation: Only the energy condition is applied and no temperature gating is performed.
- Proposed scheme: Joint temperature–energy activation with three different temperature windows, ΔT ∈{6 °C, 10 °C, 20 °C}, around the hotspot’s nominal surface temperature.

We focus on four performance metrics: (i) the total throughput *S* defined in (18), i.e., the average number of successfully decoded packets per frame; (ii) the effective hotspot throughput $\Theta_{H}$ in (27), i.e., the number of successfully decoded packets originating from hotspot nodes; (iii) the hotspot task completion time, defined as the expected number of frames required to collect a target number of hotspot packets; and (iv) the energy per effective hotspot packet, defined as the average transmit energy used by the UAV divided by the number of successfully decoded hotspot packets.

A successfully received packet is counted as an *effective hotspot packet* if the sender’s ground-truth temperature falls within a tolerance interval around the target center temperature $T_{c}$:

(28) $$T_{i}\in \left[T_{c}-\Delta_{\mathrm{eval}}/2,\;T_{c}+\Delta_{\mathrm{eval}}/2\right],$$

where $\Delta_{\mathrm{eval}}$ denotes the evaluation width. In our simulations, $\Delta_{\mathrm{eval}}$ = 6 °C (i.e., ± 3 °C), which reflects a practical detection tolerance while making the impact of sensing errors observable.

### 4.1. Impact of Node Density on Total Throughput

**Figure 2:**
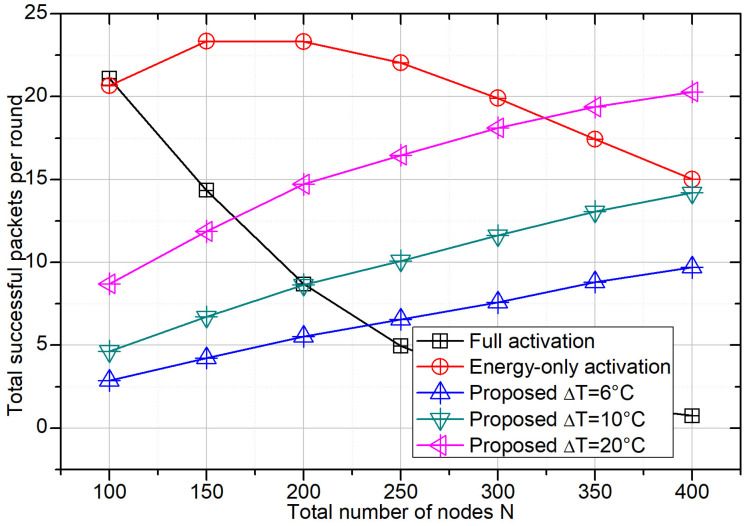
Total throughput (successful packets per frame) versus the total number of nodes *N* under different activation schemes. Full activation: all nodes contend. Energy-only: nodes contend only if $E_{i}\;\geq \;E_{\mathrm{th}}$. Proposed: nodes contend only if $E_{i}\;\geq \;E_{\mathrm{th}}$ and the measured temperature falls within a UAV-broadcast window centered at $T_{c}$ with width ΔT (i.e., $\left[T_{c}-\Delta T/2,\;T_{c}+\Delta T/2\right]$).

Figure 2 shows the average number of successfully decoded packets per frame versus the total number of nodes *N* under different activation mechanisms. With full activation, throughput is high in sparse networks but decreases rapidly as *N* grows, because nearly all nodes contend in every frame, and collisions dominate once the number of contenders far exceeds the slot number *M*. Energy-only activation mitigates collisions by limiting the active set through the energy threshold, and it therefore maintains relatively stable throughput over a wide range of node densities. In contrast, the proposed temperature-aware scheme increases throughput with *N* while keeping contention controlled, since additional nodes mainly contribute within the hotspot-relevant subset admitted by the temperature window. A wider window (e.g., ΔT = 20 °C) admits more contenders after energy gating and yields higher total throughput, at the cost of reduced selectivity because more non-core-hotspot nodes can also be activated.

### 4.2. Effective Hotspot Throughput

**Figure 3:**
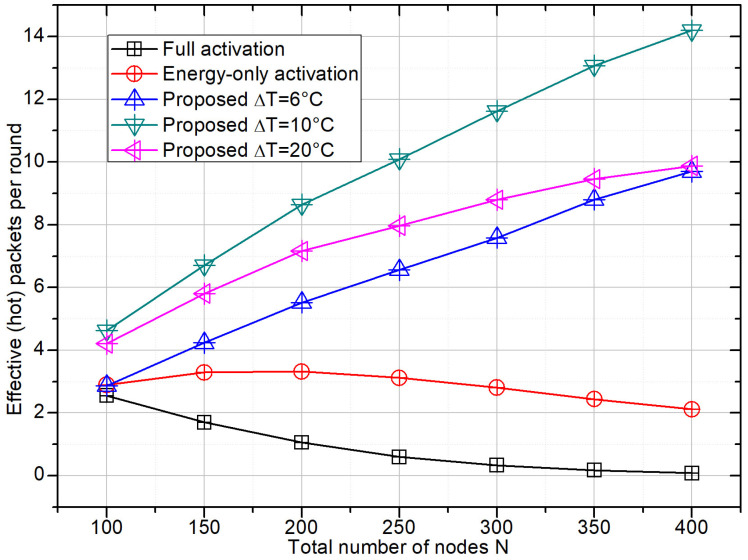
Effective hotspot throughput (successful packets per frame originating from hotspot nodes) versus *N*. For evaluation, a packet is counted as an *effective hotspot* if its transmitter satisfies the ground-truth temperature criterion $T_{i}\in [T_{c}\;-$ 3 °C,$\;T_{c}$ + 3 °C]. The proposed scheme uses an activation window of width ΔT centered at $T_{c}$, where ΔT controls how aggressively the UAV filters contenders in the temperature domain.

Figure 3 focuses on the effective hotspot throughput, i.e., the average number of successfully decoded packets originating from hotspot nodes. Here the advantage of the proposed scheme is more pronounced. Under full activation, the majority of successful packets are contributed by nodes that are thermally normal, so the effective hotspot throughput remains very low and even decreases with *N* because additional nodes mainly generate interference. The energy-only activation provides a slight improvement by suppressing far-away nodes that harvest insufficient energy, but its effective hotspot throughput still saturates quickly.

By contrast, the proposed scheme significantly boosts the number of hotspot packets: even with a relatively narrow temperature window ΔT = 6 °C, the effective throughput grows steadily with *N*; for ΔT = 10 °C it can be more than four times that of the baselines at high node densities. The curve for ΔT = 20 °C lies between the other two proposed configurations, reflecting the trade-off between activating more marginal nodes and introducing additional contention. This demonstrates that shrinking the contention set in the temperature domain is an efficient way to concentrate channel resources on geothermally relevant sensors.

### 4.3. Hotspot Task Completion Time

**Figure 4:**
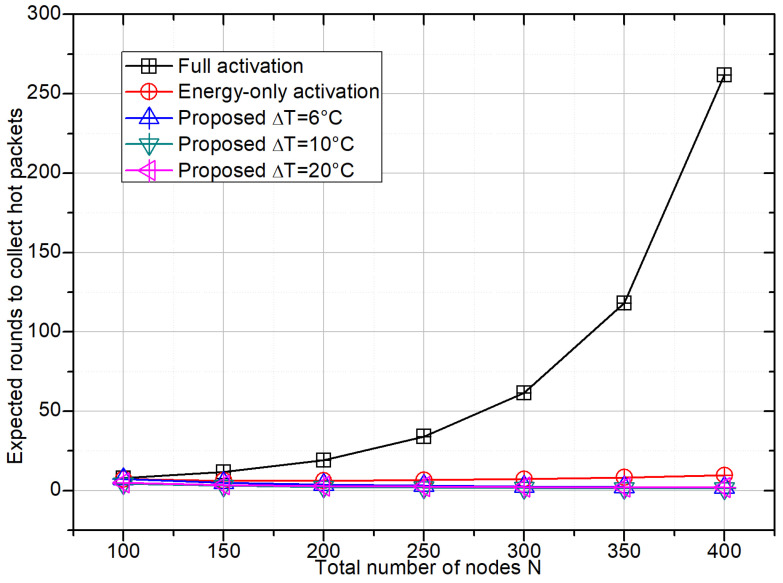
Hotspot task completion time versus *N*, defined as the expected number of frames required to collect $N_{\mathrm{hot}}=20$ effective hotspot packets. Effective hotspot packets follow the same ground-truth criterion as in Figure 3.

Figure 4 reports the expected number of frames required to collect a fixed number of hotspot packets as a function of *N*. Because the full-activation scheme produces very few hotspot packets per frame, its task completion time grows sharply with node density and becomes prohibitive when *N* exceeds 300. The energy-only activation performs somewhat better, but its completion time still slowly increases because the fraction of hotspot packets among all successful packets decreases with *N*.

In contrast, all configurations of the proposed temperature-aware scheme yield completion times close to a few frames and are almost insensitive to node density. Among them, the ΔT = 10 °C configuration achieves the best compromise, reaching the target number of hotspot packets in fewer than five frames even at N=400. This demonstrates that the proposed access mechanism can provide predictable sensing latency for hotspot monitoring, whereas conventional activation strategies suffer from strong densification effects.

### 4.4. Energy Efficiency for Hotspot Sensing

**Figure 5:**
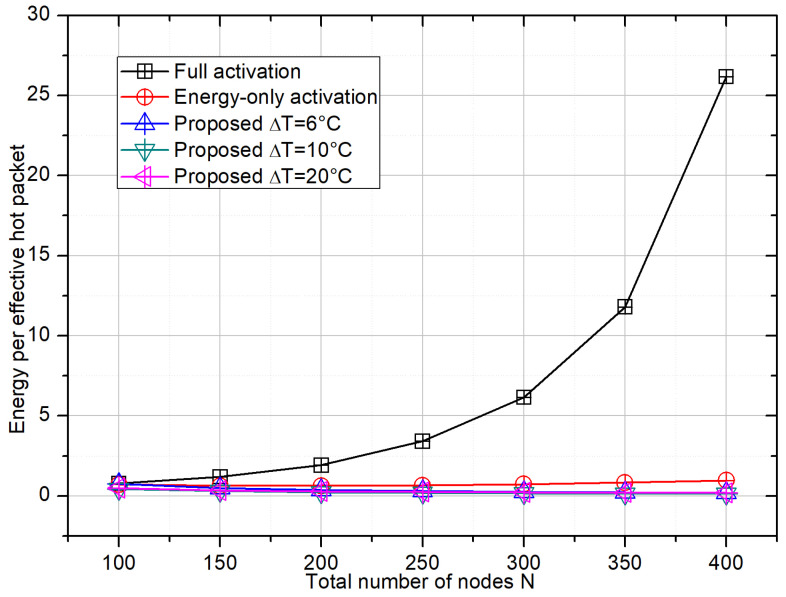
Energy cost per effective hotspot packet versus *N*. The per-frame energy is computed as $E_{\mathrm{round}}=P_{T}\tau_{w}+K_{\mathrm{act}}P_{\mathrm{node}}\left(T_{\mathrm{frame}}/M\right)$, and the number of effective hotspot packets follows the ground-truth criterion in Figure 3.

Figure 5 shows the energy per effective hotspot packet as a function of *N*. For the full-activation scheme, the UAV must repeatedly transmit energy frames while collecting only a small number of hotspot packets, leading to an energy cost that increases rapidly with node density. The energy-only activation reduces this cost but still exhibits a clear upward trend. In contrast, the proposed scheme maintains a nearly flat or even slightly decreasing energy cost as *N* grows, because higher node density mainly helps populate the hotspot region while the temperature–energy gating keeps the contention set under control. Among the three configurations, ΔT=10 °C again provides the best trade-off, achieving an improvement in energy efficiency of more than one order of magnitude over full activation at high densities.

Overall, the simulation results indicate that coupling infrared-assisted hotspot localization with temperature-window-based activation and wireless power transfer leads to substantial gains in both effectiveness and efficiency of geothermal hotspot sensing, especially in dense deployments where conventional activation schemes become interference-limited.

### 4.5. Robustness to IR/Temperature Measurement Uncertainty

To evaluate robustness to temperature sensing uncertainty, the temperature used for activation is modeled as $T_{i}^{\mathrm{obs}}=T_{i}+\epsilon_{i}$, where $\epsilon_{i}∼N\left(0,\sigma_{\mathrm{IR}}^{2}\right)$. The observation $T_{i}^{\mathrm{obs}}$ is used only for the temperature-window activation decision, while hotspot membership for evaluation is determined by the ground-truth temperature $T_{i}$. An effective hotspot packet is counted if $T_{i}\in \left[T_{c}-3\;{}^{\circ}C,\;T_{c}+3\;{}^{\circ}C\right]$. We also report the energy cost per effective hotspot packet, where the per-round energy is $E_{\mathrm{round}}=P_{T}\tau_{w}+K_{\mathrm{act}}P_{\mathrm{node}}\left(T_{\mathrm{frame}}/M\right)$.

**Figure 6:**
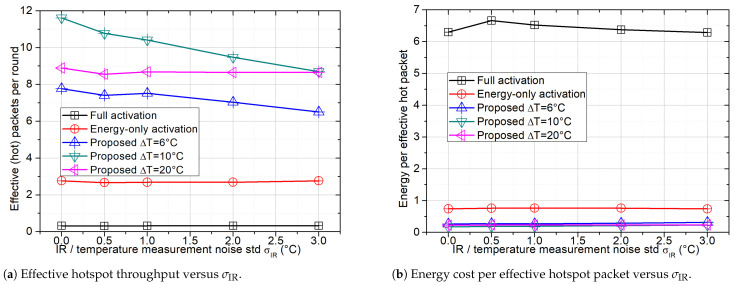
Impact of temperature/IR measurement noise on task effectiveness and energy cost (N=300). The proposed scheme applies temperature-window activation based on $T_{i}^{\mathrm{obs}}$, with window width ΔT centered at $T_{c}$; thus, larger ΔT corresponds to a less selective filter (more contenders admitted), while smaller ΔT enforces stricter hotspot focusing but is more sensitive to measurement noise.

Figure 6a shows that the proposed temperature-window activation degrades as $\sigma_{\mathrm{IR}}$ increases, due to activation misclassification (missed hotspot nodes and spurious non-hotspot activations). The degradation is more pronounced for a narrower window (e.g., ΔT=6 °C), whereas a wider window (e.g., ΔT=20 °C) remains more robust. Baselines that do not rely on temperature-based activation are largely insensitive to $\sigma_{\mathrm{IR}}$, which is consistent with the error model.

Figure 6b reports the energy cost per effective hotspot packet. Full activation incurs the highest cost because it activates the largest set of contenders, leading to excessive uplink attempts and contention while producing relatively few effective hotspot packets. By filtering contenders, the proposed schemes substantially reduce the energy cost; the advantage diminishes for narrow windows at larger $\sigma_{\mathrm{IR}}$.

## 5. Conclusions

This paper investigated an infrared-assisted, temperature-aware UAV-based sensing framework for geothermal hotspot monitoring using low-power backscatter sensor networks. By leveraging onboard thermal infrared imaging, the UAV is able to obtain a coarse thermal overview of the monitored area and selectively focus its sensing and data collection effort on regions exhibiting elevated surface temperature. A lightweight two-stage activation mechanism was designed, in which sensor nodes decide whether to participate in backscatter transmission based on both harvested RF energy and local temperature measurements. This joint temperature–energy criterion naturally suppresses transmissions from thermally normal regions while concentrating sensing resources on geothermal anomalies, without requiring complex coordination or per-node scheduling.

To better reflect the objectives of geothermal monitoring tasks, hotspot-oriented performance metrics were introduced, including effective hotspot throughput, hotspot task completion time, and RF energy cost per effective hotspot report. Simulation results under randomly generated geothermal-like temperature fields demonstrated that the proposed temperature-aware access mechanism can significantly improve sensing efficiency and reduce unnecessary energy expenditure compared with conventional full-activation and purely energy-based schemes. The results further revealed an inherent trade-off between aggressive filtering of irrelevant nodes and channel utilization efficiency, providing practical guidance for selecting temperature windows and energy thresholds in real deployments.

Overall, this study highlights the potential of integrating aerial thermal sensing with energy-aware backscatter communication to enable efficient, targeted, and scalable geothermal hotspot monitoring. The proposed framework is particularly suitable for energy-constrained, sparsely instrumented environments and can be extended to other temperature-driven sensing applications such as wildfire surveillance, industrial heat monitoring, and environmental anomaly detection.

## 6. Future Work and Practical Validation

Future work will focus on validating the proposed infrared-assisted access control under realistic sensing and radio conditions, to bridge the gap between the current modeling study and field deployment in geothermal monitoring. First, the synthetic geothermal-like temperature fields can be replaced by thermal maps derived from real UAV-borne TIR surveys or public geothermal datasets (e.g., Landsat/ECOSTRESS LST products), allowing the target temperature window to be extracted from measured land surface temperature with standard radiometric calibration, atmospheric correction, and emissivity adjustment steps. Second, the impact of TIR estimation uncertainty can be further quantified by introducing temperature-window bias and variance consistent with practical emissivity variations and atmospheric correction errors, which will enable precise evaluation of hotspot miss-detection and false-activation rates in real scenarios. Third, a small-scale prototype testbed is feasible using a commercial rotary-wing UAV (equipped with a thermal camera and an RF transmitter/reader) and battery-less backscatter tags integrated with temperature sensors. This testbed will verify the end-to-end workflow of “TIR reconnaissance → target window broadcasting → energy-harvesting-assisted backscatter collection,” and validate the performance gains of the proposed temperature–energy gating mechanism in practical environments. Additionally, the proposed framework can be extended to incorporate UAV trajectory optimization and dynamic temperature-window adjustment based on real-time TIR feedback, further enhancing its adaptability to complex geothermal terrains.

## Data Availability

The original contributions presented in this study are included in the article. Further inquiries can be directed to the corresponding author.
